# Degradation of Emerging Plastic Pollutants from Aquatic Environments Using TiO_2_ and Their Composites in Visible Light Photocatalysis

**DOI:** 10.3390/molecules30153186

**Published:** 2025-07-30

**Authors:** Alexandra Gabriela Stancu, Maria Râpă, Cristina Liana Popa, Simona Ionela Donțu, Ecaterina Matei, Cristina Ileana Covaliu-Mirelă

**Affiliations:** 1 Biotechnical Systems Engineering Doctoral School, National University of Science and Technology Politehnica Bucharest, 313 Splaiul Independentei, 060042 Bucharest, Romania; stancualexandragabriela@gmail.com; 2 Faculty of Materials Science and Engineering, National University of Science and Technology Politehnica Bucharest, 060042 Bucharest, Romania; ecaterina.matei@upb.ro; 3 National Institute of R&D for Optoelectronics INOE 2000, Atomistilor 409, 077125 Magurele, Romania; cristina.popa@inoe.ro (C.L.P.); simona.dontu@inoe.ro (S.I.D.); 4 Faculty of Biotechnical Systems Engineering, National University of Science and Technology Politehnica Bucharest, 313 Splaiul Independentei, 060042 Bucharest, Romania; cristina_covaliu@yahoo.com

**Keywords:** micro/nanoplastic, TiO_2_, visible light photocatalysis, degradation, aquatic environment

## Abstract

This review synthesized the current knowledge on the effect of TiO_2_ photocatalysts on the degradation of microplastics (MPs) and nanoplastics (NPs) under visible light, highlighting the state-of-the-art techniques, main challenges, and proposed solutions for enhancing the performance of the photocatalysis technique. The synthesis of TiO_2_-based photocatalysts and hybrid nanostructured TiO_2_ materials, including those coupled with other semiconductor materials, is explored. Studies on TiO_2_-based photocatalysts for the degradation of MPs and NPs under visible light remain limited. The degradation behavior is influenced by the composition of the TiO_2_ composites and the nature of different types of MPs/NPs. Polystyrene (PS) MPs demonstrated complete degradation under visible light photocatalysis in the presence of α-Fe_2_O_3_ nanoflowers integrated into a TiO_2_ film with a hierarchical structure. However, photocatalysis generally fails to achieve the full degradation of small plastic pollutants at the laboratory scale, and its overall effectiveness in breaking down MPs and NPs remains comparatively limited.

## 1. Introduction

The pollution generated by microplastics (MPs) and nanoplastics (NPs) (plastic particles typically smaller than 5 mm and 1 µm, respectively) is considered irreversible and widespread, posing significant threats to ocean health [1], coastal tourism [2,3,4,5,6,7,8,9,10], wildlife, food safety [11,12,13,14], soil [15,16,17], and climate regulation [18,19,20]. Due to their large surface to volume ratio and chemical surface properties, MPs and NPs can adsorb other chemical pollutants from the aquatic environment, which could be transferred to marine and freshwater organisms [21,22,23,24]. MPs/NPs enter the human body through the consumption of food, tap water, and bottled water [14,25], inhalation from air, and even dermal contact [26]. PVC, PE, PS, and ABS are among the most commonly used plastics worldwide, contributing significantly to the generation of both MPs and NPs [27,28]. Synthetic fabrics are a dominant contributor to MP pollution, with plastic filaments from clothing made of polyester and polyamide microfibers being the most prevalent form of plastic particles found in wastewater [29,30]. Among all plastic types, polystyrene (PS) and polyvinyl chloride (PVC) are more prevalent MPs and NPs identified in human implants [31]. NPs were found within the placenta [32]. The potential human health risk of these emerging plastic pollutants is well documented [26,33,34,35,36]. According to the Agency for Toxic Substances and Disease Registry (ATSDR), these substances are associated with carcinogenesis [31]. The long-term impacts of MPs and NPs on the environment and human health underscore the pressing need for global actions to address this pervasive form of plastic pollution [37].

While traditional methods such as mechanical and chemical recycling have been employed to manage plastic waste, they are not well suited for the effective disposal of MPs and NPs. This limitation is primarily due to the challenges posed by the small size and complex composition of these emerging plastic pollutants. Consequently, alternative approaches, including physical separation and degradation techniques, have been suggested for the removal of MPs and NPs from aquatic environments [38,39,40,41,42,43].

The physical separation of MPs and NPs encompasses adsorption, coagulation/flocculation combined with sedimentation, filtration, flotation, membrane bioreactors (MBRs), and magnetic separation techniques [44,45,46,47,48,49,50,51]. Among these, adsorption has gained significant attention due to its numerous advantages, including cost-effectiveness, ease of implementation, the versatility of adsorbent materials, and broad adaptability [51,52,53,54]. Chen et al. [28] reported remarkable adsorption capacities for various commercial MPs ranging from 2000 µm to 100 µm in their sizes, such as PP, PE, PVC, PP, PS, and ABS in highly saline water of 60.2 mg/g, 67.4 mg/g, 58.6 mg/g, 30.3 mg/g, 58.6 mg/g, 43.2 mg/g, and 36.1 mg/g, respectively, using an engineered green MOF-based super hydrophobic sponge. A drawback of adsorption is the difficulty in recycling the sorbents used in the process [50]. The complete removal of MPs through coagulation/flocculation, sedimentation (CFS), and filtration processes has been observed for particles larger than 10 µm [55]. For smaller particles (<1 µm), removal rates exceeding 80% have been achieved [55]. Optimizing water treatment conditions is expected to further improve the efficiency of MP removal from water systems [4]. However, the study of NP exposure presents greater challenges due to their even smaller particle sizes. The high NP sizes (200 nm) are almost entirely removed through CFS processes, whereas smaller NPs (50 nm) are most effectively eliminated using granular activated carbon (GAC) filtration [54,56]. Additionally, ionic liquids have been used to separate NPs from drinking water [57]. Filtration can be tailored and adjusted to fit different scales and needs, and it can be used to effectively separate MPs from a variety of water sources, such as drinking water treatment systems and wastewater treatment plants [51]. The removal efficiency of these methods depends on several factors, including particle type, size, surface charge, water properties, and operating conditions [58]. However, the separation methods for treating MPs/NPs face several challenges such as irreversible membrane fouling [45], filter clogging, the potential release of MPs/NPs into drinking water supply networks during membrane filtration, and the routine cleaning and maintenance required for sedimentation tanks [51,59].

**Figure 1:**
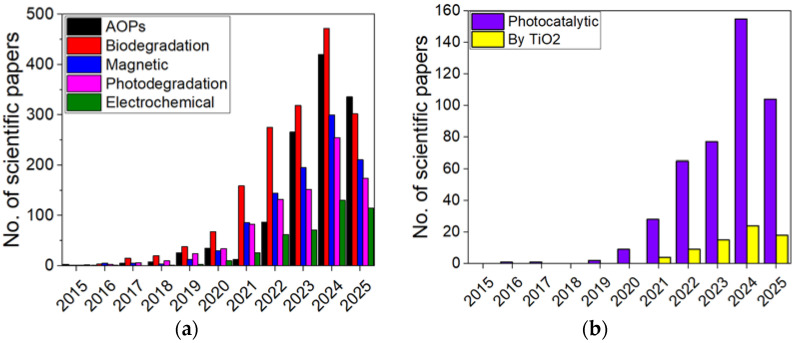
The number of papers published from 2015 up to now on degradation of MPs and NPs according to ScienceDirect platform. (**a**) Different degradation methods; (**b**) photocatalytic degradation. Key words: “degradation of micro and nanoplastics”, “advanced oxidation processes”, “biodegradation”, “photodegradation”, “magnetic degradation”, “electrochemical degradation”, “photocatalysis”, “photocatalysis by TiO_2_”.

Common degradation methods for MPs and NPs include advanced oxidation processes (AOPs), electrochemical techniques, biodegradation, bioremediation, photodegradation, and magnetic degradation [50,60,61]. Figure 1 shows the number of scientific papers published annually from 2015 to 2025 (up to the current data) on the various methods of MP and NP degradation.

There is a clear upward trend in the number of publications for all methods, with a dominance of AOPs and biodegradation, indicating increasing research interest in the degradation of MPs and NPs. The growth is particularly pronounced after 2020, suggesting an intensification of global efforts to address this environmental challenge (Figure 1a). Ozonation, as part of AOPs, has shown promise for addressing NP pollution in drinking water treatment plant (DWTP) facilities. For example, the application of ozonation to PS NPs in a DWTP achieved a 99.9% reduction in molecular weight and 42.7% mineralization within 240 min [62]. The degradation products included formic acid, phenol, acetophenone, hydroquinone, and various other compounds [62]. Highly effective strategies such as mycoremediation, the use of soil microbes to enhance biodegradation, and the phytoextraction of MPs have shown potential but come with significant toxicity risks. Moderately effective methods, including plant-assisted remediation, rhizosphere degradation, phytodegradation, and biodegradation, achieve effectiveness rates of 50% to 65% with moderate toxicity risks [41]. El-Kurdi et al. [63] introduced a promising approach to PS NP bioremediation by isolating five bacterial strains from the gut microbiome of *Tenebrio molitor* larvae fed on different plastic diets. Their study demonstrated a 92.3% reduction in PS NP size (from 0.3 µm to 0.02 µm) and a 7% weight loss over 30 days. While these methods show potential, they are not without notable challenges. The mechanisms underlying MP biodegradation remain poorly understood, and the process is typically slow. Furthermore, the effective implementation of biodegradation is costly, requiring the isolation of efficient microbial strains and the cloning of specific enzymes capable of breaking down MPs [64,65]. Another concern is the formation of toxic and hazardous volatile by-products during the biodegradation process, raising questions about potential secondary environmental impacts. Although degradation approaches offer a more sustainable alternative, extensive research and optimization are necessary to overcome these obstacles and enhance their feasibility for large-scale applications. Notably, biodegradation is not well suited for removing non-hydrolyzed MPs, further limiting its effectiveness.

Among advanced oxidation processes (AOPs), photocatalytic degradation has gained attention as a promising green method for the removal of MPs and NPs from water environments [64,66,67,68,69,70]. This environment friendly technology offers several advantages, including a low cost, environmental sustainability, and high efficiency in mineralizing plastic pollutants [64]. One of the most innovative and state-of-the-art solutions for plastic removal is photoreforming. This new approach involves the use of photocatalysis with the aim of achieving two major goals: the degradation of plastic waste and the production of hydrogen fuel and other valuable chemicals [71,72,73,74].

Titanium dioxide (TiO_2_) is one of the most widely studied and used materials for pollutant degradation in photocatalysis under UV and VIS sources [28,30,75,76,77,78]. Since Honda and Fujishima’s seminal discovery of solar photocatalytic water splitting on TiO_2_ electrodes in 1972, photocatalysis has been rigorously explored, ushering in a transformative era for sustainable energy conversion applications [64].

Domínguez-Jaimes et al. [75] explored the degradation of PS NPs in water with TiO_2_ with tubular and barrier shapes. A fluoride-doped TiO_2_ (F-TiO_2_) photocatalyst working under UV light was created for the remediation of a PE polymer from wastewater treatment plants [79]. Garcia-Munoz et al. [76] created TiO_2_-P25/β-SiC foams containing commercially available powdery Aeroxide TiO_2_ P25 obtained from Evonik (Germany) for PS nanobead degradation in water. Chen et al. [28] successfully synthesized a green engineering TiO_2_/Ni-MOF-based super hydrophobic sponge for the in-situ adsorption of various MPs and the photocatalytic degradation of pesticides from high-salinity water. However, the photocatalytic efficiency of TiO_2_ is limited due to its intrinsic bandgap of 3.2 eV, which confines its activity to the UV spectrum, thereby reducing its effectiveness under natural sunlight for visible light-driven applications [73]. Furthermore, its efficiency is hindered by the high recombination rate of photoexcited electron–hole (e^−^/h⁺) pairs, which minimizes the availability of charge carriers for redox reactions. Additionally, the relatively low surface area of TiO_2_ reduces its interaction with pollutants, thereby limiting its catalytic performance [77]. These limitations signify that it is impossible to use solar light for photocatalysis in a large-scale experiment. Even under UV irradiation, the ability of TiO_2_ to break down some of the most prevalent plastic structures (PE, PP, PVC, PS) is limited [80,81]. It was proven that in the case of PP films, simple TiO_2_ decomposition efficiency under xenon lamp irradiation was insignificant [82]. These findings emphasize the need to use TiO_2_-based composites for better results involving plastic degradation.

Figure 1b indicates that the number of TiO_2_-focused studies in the MP and NP domain remains a small fraction of the total photocatalytic research, which has risen sharply since 2020, with significant peaks in 2023 and 2024. However, this photocatalyst material is an indispensable material for photocatalytic applications [30].

This review synthesized the current knowledge on the effect of TiO_2_ photocatalysts on the degradation of MPs and NPs under visible light, highlighting the main results, challenges, and solutions for enhancing the performance of the photocatalysis technique. It is structured into five sections: Section 1, Introduction—highlights the advantages and disadvantages of conventional methods used for the elimination of MPs and NPs; Section 2, Characteristics of TiO_2_ and their Composites for the Photocatalytic Degradation of MPs and NPs —discusses the synthesis and characterization of TiO_2_ composites for the photocatalytic degradation of emerging plastic pollutants and explains the principle of photocatalysis under visible light; Section 3, Performance of TiO_2-_Based Photocatalysts for MP/NP Degradation under Visible Light Irradiation—focuses on the photocatalytic degradation of MPs and NPs in aquatic environments under visible light, including the underlying mechanisms; Section 4, Challenges—discusses the challenges faced in this area, while Section 5, Conclusions—concludes with insights and future prospects.

To compile relevant findings for this review, the scientific literature concerning MP and NP treatment, photocatalysis, and TiO_2_ were extensively examined. This process included a comprehensive search using databases such as Google Scholar, Elsevier, SpringerLink, and ScienceDirect. The keywords used in this search included “TiO_2_”, “nanoplastics” “microplastics (MPs)”, and “photocatalytic degradation of MPs/NPs by TiO_2_ in Visible”. A wide range of studies were carefully analyzed to ensure the inclusion of pertinent data.

## 2. Characteristics of TiO_2_ and Their Composites for the Photocatalytic Degradation of MPs and NPs

### 2.1. Principle of Photocatalysis

**Figure 2:**
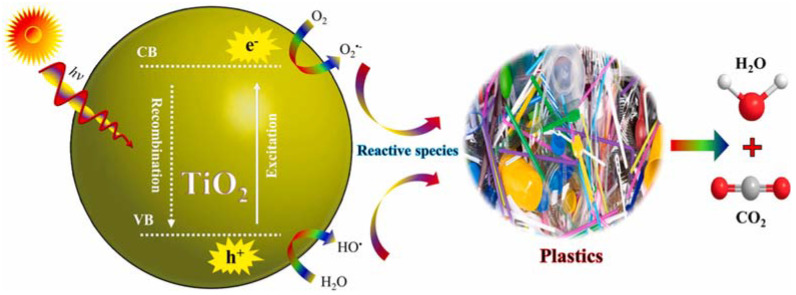
Degradation of MPs and NPs through photocatalysis under visible light. Reproduced from [77] with permission from Elsevier.

The photocatalytic degradation of plastic pollutants occurs through two primary mechanisms: direct degradation and indirect degradation [66]. The photocatalytic breakdown of MPs and NPs with a TiO_2_ semiconductor involves three key stages: (i) the absorption of photons with energy hν ≥ bandgap (Eg), initiating semiconductor activation; (ii) the movement and separation of photogenerated electrons (e^−^) and holes (h^+^), with electrons moving to the conduction band (CB) and leaving positive holes in the valence band (VB); (iii) redox reactions at the semiconductor surface, where e^−^ and h^+^ react with adsorbed water (H_2_O) and oxygen (O_2_), producing reactive oxygen species (ROS) like hydroxyl (OH^●^), hydroperoxy (HOO^●^), and superoxide (O_2_^●−^) radicals [51,64,83]. These ROS facilitate the degradation of MPs and NPs by breaking chemical bonds and altering their structure into smaller molecular chains [30,84], eventually mineralizing them into carbon dioxide (CO_2_), water (H_2_O), and simple organic compounds (Figure 2).

The indirect mechanism, which is the dominant pathway, involves five distinct stages (according to Equations (1)–(6): excitation (Equation (1)), water ionization (Equation (2)), the formation of superoxide radicals (Equation (3)), and superoxide protonation (Equation (4)). In contrast, direct degradation (represented by Equations (7)–(9)) is less effective compared with the indirect mechanism, leading to small chemical compounds.

(1) $${TiO}_{2}\to^{h_{VB}^{+}+\;e_{CB}^{-}}$$

(2) $$h_{VB}^{+}+H_{2}O\to {HO}^{•}+H^{+}$$

(3) $$O_{2}+e_{CB}^{-}\to O_{2}^{•-}$$

(4) $$O_{2}^{•-}+H^{+}\to {HOO}^{•}$$

(5) $${2\;HOO}^{•}\to O_{2}+H_{2}O_{2}$$

(6) $$H_{2}O_{2}\to {2HO}^{•}$$

(7) $$h_{VB}^{+}+MPs\;or\;NPs\to Oxidized\;Product$$

(8) $${HO}^{•}+MPs\;or\;NPs\to {CO}_{2}+H_{2}O$$

(9) $$e^{-}+MPs\;or\;NPs\to Reduced\;Product$$

### 2.2. Strategies for the Photocatalytic Degradation in Visible Light

If visible light is used for irradiation, the photocatalytic efficiency risks decreasing even further. Scientists have found a solution to this issue, by engineering the bandgap through doping, creating hybrid junctions, and surface functionalization, thus being able to extend the absorption wavelength range to the visible light domain [85,86,87].

The modification of TiO_2_, such as its coupling with other materials (e.g., graphene oxide (GO) or metal/non-metal nanoparticles) is commonly employed [27]. Nabi et al. [77] have shown, using SEM images, that doping TiO_2_ with vitamin C enhances the compound ability to form cavities in the plastic material, specifically PVC and PE. This approach reduces the bandgap, improves visible light absorption, and promotes the separation of photoexcited charge carriers, thereby boosting photocatalytic efficiency.

The combination of p-type and n-type semiconductors to form a heterojunction generates a built-in electric field that promotes the effective separation of e^−^/h^+^ pairs. The concept of surface heterojunctions introduces another innovative strategy for designing and developing photocatalysts by leveraging the variations in the energy band structure of different crystalline surfaces. This spatial separation of electron–hole pairs, coupled with optimized redox potentials, enhances sunlight absorption and increases the number of surface-active sites. Together, these factors significantly boost the photocatalytic performance of TiO_2_ [88].

### 2.3. Synthesis of TiO_2_-Based Photocatalysts

Table 1 summarizes the modification routes of TiO_2_ and the main characteristics achieved for exploiting the photocatalytic degradation of different MPs and NPs under visible light in aquatic media.

The methods used for the preparation of TiO_2_-based composites are sol–gel [27,38,89,90,91], solvothermal [30,92,93], evaporation-induced self-assembly (EISA) [94], and anodic oxidation approaches [95].

The green synthesis of a N-TiO_2_ photocatalyst was carried out using a pore-forming agent and a nitrogen precursor such as a cleared extrapallial fluid of fresh blue mussels (*Mytilus edulis*) and a tri-block copolymer (Pluronic^®^ F-127) [89].

The C,N-TiO_2_/SiO_2_ photocatalysts were synthesized using extrapallial fluid extracted from two mussel species: *Mytilus edulis* (designated as TS-ME) and *Mytilus galloprovincialis* mussels (referred to as TS-MG) [93].

The sol–gel method involves a precursor such as titanium(IV) butoxide, TiCl_4_ [38,91], or tetrabutyl titanate [90]. In all approaches, calcination at higher temperatures is required.

Heterogeneous photocatalysis using the coupled catalyst CuO/TiO_2_ derived from the bimetallic HKUST-1(Cu/Fe) was prepared using the solvothermal method. First, a MOF, HKUST-1 (Hong Kong University of Science and Technology), with different colors due to the amount of copper and iron oxides, was prepared using the solvothermal method, followed by calcination, and deposited on borosilicate glass. Then, the HKUST-1(Cu/Fe)/TiO_2_ composites containing 5, 10, and 15 wt % of MOF were prepared using titanium (IV) butoxide and ethanol/acetic acid solution and a treatment at 180 °C for 18 h with a heating ramp of 5 °C min^−1^ in a Teflon reactor [30].

An innovative approach developed by Xue et al. [95] involved doping α-Fe_2_O_3_ nanoflowers onto a TiO_2_ film with a hierarchical structure comprising an inverse opal-like layer and nanotube arrays (denoted as α-Fe_2_O_3_/TiO_2_ HNTAs), using a two-step anodic oxidation process. The deposition of α-Fe_2_O_3_ nanoflowers was performed via a hydrothermal method for 1 h, 2 h, and 3 h. The sample with a 3 h deposition time exhibited enhanced absorbance up to 700 nm.

The synthesis methods for enhancing the photocatalytic performance of TiO_2_ often involve bandgap engineering through doping and the formation of heterojunctions.

Doping involves incorporating foreign atoms into the TiO_2_ lattice to modify its electronic structure, often achieved via the sol–gel method. Non-metal doping with elements such as N [27,89], C [38], graphene oxide (GO) [27], or g-C_3_N_4_ [90] introduces mid-gap states that enable visible light absorption by reducing the bandgap energy. This strategy enhances TiO2 photocatalytic performance under visible light.

Llorente-García et al. [94] designed a mesoporous N-TiO_2_ coating for the photocatalytic degradation of HDPE and LDPE MPs using the evaporation-induced self-assembly (EISA) technique. In this process, 9.1 × 10^−^^5^ mol of Pluronic^®^ F127 was dissolved in 0.728 mol of absolute ethanol. Subsequently, 0.0182 mols of TiCl_4_ was gradually added along with 0.182 mol of deionized water and 0.0025 mol of urea. After stirring the mixture for 5 min, the coating was applied onto pre-cleaned glass substrates via dip-coating at an immersion and withdrawal speed of 100 mm/min under 45% relative humidity. The resulting mesoporous coating underwent thermal treatment at 200 °C for 24 h to stabilize the mesoporous framework and at 500 °C for 3 h to promote the crystallization of anatase-phase TiO_2_.

Ariza-Tarazona et al. [38] successfully synthesized a C,N-TiO_2_ bio-inspired photocatalyst by exploiting the C and N sources from the extrapallial fluid (EPF) of *Mytilus edulis* mussels offering a promising strategy to enhance the photocatalytic performance under visible light. C doping enhances light absorption by forming hybridized states near the conduction and valence bands, while N doping contributes to narrowing the bandgap by creating localized states that enable visible light activation.

C,N-TiO_2_/SiO_2_ photocatalysts combining both doping (C, N into TiO_2_) and heterojunction properties (TiO_2_ with SiO_2_) were accomplished by Ariza-Tarazona et al. [93]. The inclusion of C and N in TiO_2_ involves the substitution or interstitial incorporation of these elements into the TiO_2_ lattice, thus altering its electronic band structure and enabling visible light activity, when the TiO_2_/SiO_2_ combination forms a heterojunction.

**Table 1:** Modified TiO_2_ photocatalysts and their characteristics for the photocatalytic degradation of MPs/NPs under VIS light.

Semiconductor	Method	Main Characteristics	Plastic Pollutant/Solution for Degrading	Photocatalysis Conditions	Efficiency	Ref.
TiO_2_ NPs	Sol–gel	Bandgap of 2.93 eV. Size dimension ranged from 97.93 ± 145.79 nm (SEM). Anatase phase (XRD).	PP MPs/ A ratio of 1:1 between MPs to photocatalyst. This solution was added into buffer (pH∼3, 0.4% *w*/*v* dispersion of components)	Solar irradiation (pH 3 and 50 h); average light intensity of 5 kWh/m^2^	Mass loss of 50.5 ± 0.5%	[91]
N-TiO_2_	I. Green synthesis; II. Sol–gel method	Protein-derived TiO_2_ powder was more amorphous than the sol–gel synthesized N-TiO_2_ (XRD). The diameters were in the range of 220–920 nm (FE-SEM).	HDPE MPs/ 2 mg/mL of the extracted MPs in distilled water	Room temperature for 20 h, with samples placed at 120 mm distance of a 27 W fluorescent lamp after 8 h of irradiation with constant light emissions in the visible spectrum (400–800 nm)	Mass loss of 6.4% in aqueous environment	[89]
C,N-TiO_2_	Sol–gel; Solvothermal	Crystallite size of 4.92 nm. Eg of 2.9 eV. Surface area of 219.42 ± 1.82 m^2^/g (FEG-SEM micrographs).	Primary HDPE MPs	Irradiation at 428 nm, photocatalysis time of 50 h with continuous stirring at 300 rpm; 50 W LED lamp; absorbance at 428 nm; pH 3, 7 and 11, and temperature of 0, 20 and 40 ± 2 °C	Mass loss of 71.77 ± 1.88% at pH 3 and 0 °C	[38]
GO/N-TiO_2_ composites (at three different ratios 1:3, 1:1, and 3:1 *w*/*w*)	I. Sol–gel method for N-TiO_2_; II. Ultrasonication technique for composite preparation	Decrease in crystallite size from 13.69 nm (TiO_2_) to 6.13 nm, 4.35 nm, and 3.13 nm for composites (XRD). Eg in the range of 2.1–2.8 eV, 2.4–2.9 eV, and 2.6 eV for those three composites. Thermal stability increased with the N-TiO_2_ content (TGA).	PVC NPs/ 0.4 mg/mL concentration of catalyst in aqueous solution of PVC-NPs	50 mL glass beaker; natural room light conditions ( tungsten bulb and room light) at 446 nm; pH of 4, 7, and 10; irradiation durations of 30, 60, 90, 120, 150, and 180 min	98.2% removal efficiency at pH 4 for 1:3 the ratio between components	[27]
g-C_3_N_4_/TiO_2_/waste cotton-based activated carbon (WCT-AC) composite	I. Sol–gel method for TiO_2_/WCT-AC; II. High-temperature thermal polymerization method from composite preparation	Eg of the TiO_2_, g-C_3_N_4_, TiO_2_/WCT-AC and g-C_3_N_4_/TiO_2_/WCT-AC were 3.12, 2.65, 3.06 and 2.54 eV, respectively.	PE MPs/ 50 mg catalyst	VIS light irradiation provided by a 500 W xenon lamp light source (*λ* > 420 nm); photocatalysis time of 200 h, and system pH of 7.0; initial light intensity of 200 mW/cm^2^ and system temperature of 25 °C	Mass loss of 67.58%	[90]
TiO_2_/MIL-100(Fe) composites	Solvothermal/microwave methods and post-annealing technique. The mixtures were heated for 12 h at 180 °C in a 100 mL Teflon-lined stainless-steel autoclave; calcined in an air muffle furnace at 350 °C for 2 h	Spherical NPs (SEM). The particle sizes increased from 29 ± 6 nm (TiO_2_) to 54 ± 15 nm (SEM). TiO_2_ crystallite size slightly decreased from 4.0 to 3.0 nm (XRD). Eg of 2.21–2.65 eV (Tauc plots) compared with 3.03 for TiO_2_. BET of 179.0 m^2^/g compared with 128.2 m^2^/g for TiO_2__._	PET NPs/ 0.1 mg/mL PET NPs in water suspension (pre-sonicated for 30 min, pH 3) and 0.125 g/L photocatalyst	A 200 mL batch reactor under simulated sunlight (Xe lamp 300–800 nm) at an intensity of 30 W/m^2^ (5 h reaction time)	Increased CI (0.99); Reduction in the turbidity ratio (0.454); Increased TOC released (3.00 mg/L); Cavities in the NPs structure (SEM)	[92]
HKUST-1(Cu/Fe)-derived CuO/TiO_2_ (TCFH) composites containing 5, 10, and 15 wt % of MOF	Solvothermal method; Temperature of 180 °C for 18 h with a heating ramp of 5 °C min^−1^; calcined at 350 °C for 2 h	Crystallite sizes for: TCFH 95:5–53.0 nm. TCFH 90:10–54.2. TCFH 85:15–58.5 (XRD). Specific surface area for: TCFH 95:5–168.54 m^2^/g. TCFH 90:10–158.08 m^2^/g. TCFH 85:15–161.95 m^2^/g, compared with 152.09 m^2^/g for TiO_2_ (BET).	Nylon 6 MPs/ 0.2 mg/mL of nylon 6 MPs suspension	Stirring at 250 rpm and irradiated with a UV–vis Hg lamp (350–700 nm, 32.3 W/m^2^) positioned at 9 cm; ambient temperature for 5 h	TOC of 11.012 mg/L for 15% wt (Cu/Fe) HKUST-1, at pH 7	[30]
Mesoporous N–TiO_2_ coating	Evaporation-induced self-assembly (EISA)	Anatase shape (XRD). E_g_ of 3.1 eV. A thickness of the microstructure of 146 ± 3 nm. The coating had a grid-like structure composed of NPs of 12 ± 3 nm and pores with a diameter of approximately 10 nm. BET surface area of 74.7 ± 0.2 m^2^/g.	HDPE and LDPE MPs/ 0.4 wt/v% of MPs dispersion in a CH_3_COONa/CH_3_COOH buffer (pH 3)	Glass container; a 215 mm distance of dispersion with catalyst from the visible LED; irradiation with a lamp of 50 W (400–800 nm) for 50 h, with continuous stirring at 300 rpm, at room temperature	Mass losses of 0.22 ± 0.02% and 4.65 ± 0.35% for two HDPE MPs with different sizes, and 0.97 ± 0.32% and 1.38 ± 0.13% for two LDPE MPs with different dimensions; CI of 0.80 and 0.45 for HDPE MPs and 1.25 for LDPE MPs	[94]
Mesoporous C,N-TiO_2_/SiO_2_	Solvothermal method; I. Preparation of C,N-TiO_2_ semiconductor, designed TS-ME, by mineralization; II. Preparation of C,N-TiO_2_/SiO_2_ semiconductor, designed TS-MG, by thermal treatment in an autoclave	TS-ME: Eg of 2.41 eV and BET of 313 m^2^/g. TS-MG: Eg of 2.93 eV and BET of 332 m^2^/g.	Secondary PET MPs/ 1:1 (wt %) of PET MPs to photocatalyst in buffer solution with a pH 6 or 8	Batch-type glass container, 50 W LED visible light lamp, 500 W/m^2^ light irradiance; 120 h of irradiation at room temperature under 350 rpm	Mass loss values ranging from 9.35 to 16.22%	[93]
α-Fe_2_O_3_/TiO_2_HNTAs	Two-step anodic oxidation process; Hydrothermal method	TiO_2_ anatase phase. The average pore size for TiO_2_HNTAs of 120 nm. The average length of the bottom nanotube array of 4.3–4.4 μm. The average thickness for α-Fe_2_O_3_/TiO_2_HNT ranging from 260 nm to 330 nm.	PS MPs spheres/0.11% *v*/*v* of MPs in ultrapure water	Halogen lamp with a light intensity of 0.5 W/cm^2^ from 2 h, 3 h to 4 h at an induced temperature of 75 °C	100% degradation after 4 h of irradiation	[95]

Zhang et al. prepared a g-C_3_N_4_/TiO_2_/waste cotton-based activated carbon (WCT-AC) composite from g-C_3_N_4_ and waste cotton-based activated carbon (WCT-AC)-loaded TiO_2_ as precursors using a mixed high-temperature thermal polymerization method [90].

Metal–organic frameworks (MOFs) are versatile materials assembled from metal salts/clusters and organic ligands through coordination bonds, with a vast porous structure, offering great potential in adsorbing and degrading pollutants associated with water treatment [96]. This class of materials, which are characterized by their highly porous structures and high surface areas could effectively remove MPs with more than 90% [97] or even higher than 98.4% [98]. When combined with TiO_2_, they offer extended light absorption (especially visible light), improved charge separation, better pollutant adsorption, and tailored active sites for specific reactions. Examples of modified TiO_2_ semiconductors combined with MOF to improve charge separation and reduce recombination rates for plastic pollutant degradation include the coupling of TiO_2_ with MIL-100(Fe) (Materieux de l′Institut Lavoisier) [92] and the incorporation of HKUST-1 (Hong Kong University of Science and Technology) into TiO_2_ [30].

### 2.4. Characteristics of TiO_2_-Based Photocatalysts

The prepared TiO_2_-based photocatalyst materials have been characterized using various analytic techniques: X-ray diffraction (XRD) for analyzing the crystalline phase; attenuated total reflectance–Fourier transform infrared (ATR-FTIR) spectroscopy for identifying functional chemical groups; scanning electron microscopy (SEM)/field emission gun scanning electron microscopy (FEG-SEM) combined with energy-dispersive X-ray spectroscopy (EDS) for examining microstructural characterization and elemental analysis; UV–vis/NIR reflectance spectra for determining the bandgap (Eg); X-ray photoelectron spectroscopy (XPS) for analyzing the elemental composition, surface electronic states, and interaction between elements [27,90,92]; and specific surface area and pore size distribution estimated using the Brunauer–Emmett–Teller (BET) method with N_2_ physisorption analysis [38,92].

To calculate the optical bandgap energy (Eg), the Tauc’s equation was applied [27].

αhν = A(hν−Eg)^n^ (10)

where h is the Planck’s constant (6.626 × 10^−34^ J × s), ν is the frequency of the irradiating light, α is the absorption coefficient, and A is a proportionality constant. The n is related to the semiconductor type of the material [90].

The Eg is estimated by finding the intercept of the straight line of the plot of (αhν)^1/2^ or (αhν)^2^ against hν.

Modifying and doping photocatalysts can reduce the bandgap and extend the material’s light absorption range, further improving light harvesting capabilities. Therefore, increasing the TiO_2_ amount in GO/N-TiO_2_ composites’ synthesis at three weight ratios between components (1:3, 1:1, and 3:1 *w*/*w*) resulted in a decreased Eg ranging from 2.1 to 2.8 eV, indicating stronger chemical bonding between N-TiO_2_ and GO [27].

The advantage of the C,N-TiO_2_ semiconductor lies in the reduction in the bandgap from 3.2 eV for the undoped sample to 2.9 eV for the protein-derived TiO_2_ [38]. Similarly, increasing the MOF amount from 5 to 15 wt% in the TCFH synthesis reduced the bandgap energy from 3.16 to 2.83 eV [30].

The morphology, size, and composition of TiO_2_ photocatalysts are critical factors influencing the photocatalysis process. For instance, TiO_2_ in a nanotubular form exhibits a unique nanotube structure with an average pore diameter of 108.5 ± 5.7 nm [75]. This morphology enhances photons interaction during the degradation process and promotes the separation of photogenerated e^−^/h^+^ pairs due to an increase in surface defects [75]. Compared with the rutile phase, the anatase phase, characterized by its tetragonal crystal structure, exhibits superior photocatalytic activity. This behavior is attributed to its longer charge carrier lifetime and greater efficiency in generating hydrogen ions, which facilitate the reduction in photogenerated electrons [91]. Several TiO_2_-based composites have been reported to retain the anatase crystalline phase in their structure [27,77,92].

**Figure 3:**
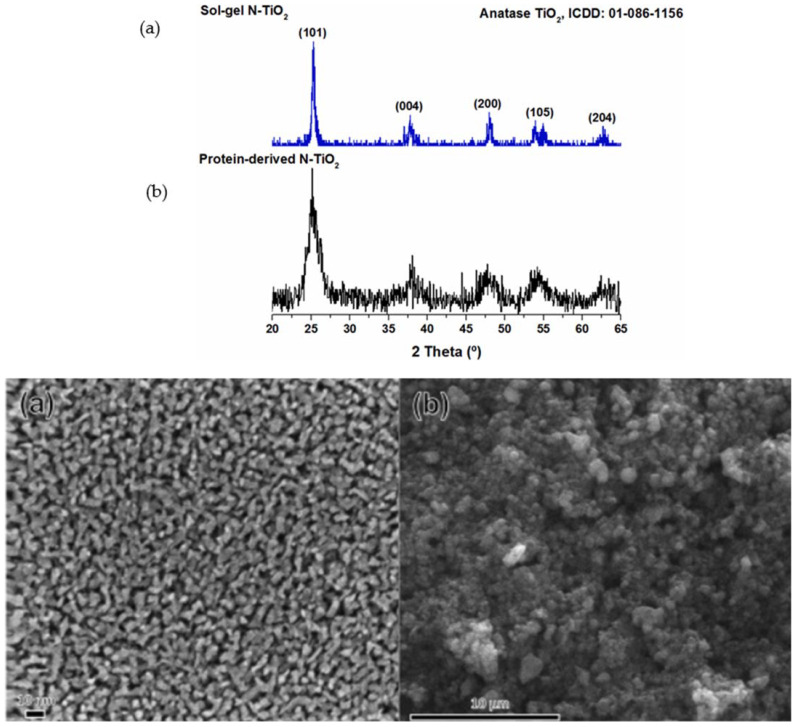
XRD patterns and FE-SEM micrographs of the sol–gel (**a**) and protein-derived N-TiO_2_ semiconductors (**b**). The two sets of labels mean XRD and SEM, respectively. Reproduced from [89] with permission from Elsevier.

N-TiO_2_ powder synthesized by sol–gel methods was more crystalline and has a uniform porosity distribution along the sample with diameters between 2 and 10 nm (Figure 3a) compared with the protein-derived TiO_2_, which contained a more amorphous phase (Figure 3b) [89]. Protein-derived N-TiO_2_ powder was characterized by a diffraction peak at circa 45°, attributable to a rutile crystalline phase (Figure 3b).

**Figure 4:**
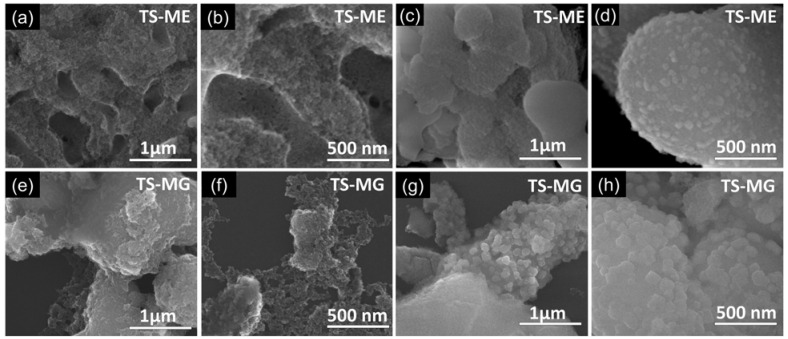
FEG-SEM micrographs of TS-ME (**a**–**d**) and TS-MG (**e**–**h**) photocatalysts. Reproduced from [93] with permission from Elsevier.

Two extrapallial fluids from *Mytilus edulis* and *Mytilus galloprovincialis* mussels, each at a concentration of 1200 μg/mL, were used as sources of C and N for the fabrication of C,N-TiO_2_/SiO_2_ photocatalysts, designated as TS-ME and TS-MG. These materials exhibited a surface area four times greater than that of a Degussa P25, demonstrating the beneficial effect of mussel-derived EPF incorporation [93]. The FEG-SEM images of both photocatalysts are shown in Figure 4. The images reveal two distinct morphologies: (1) a 3D network of TiO_2_ containing macropores with diameters ranging from 0.5 to 1.6 μm (Figure 4a,b,e,f), where proteins serves as templates to introduce porosity, (2) clusters of spherical particles (Figure 4c,d,g,h) forming agglomerates measuring 1–3 μm, which also contribute to the enhanced porosity of the C,N-TiO_2_/SiO_2_ photocatalyst [93].

Incorporating rutile into anatase has been shown to enhance the photocatalytic activity of the composite by promoting interfacial charge separation between the two phases, thereby, effectively suppressing the harmful recombination of electron–hole pairs [38].

**Figure 5:**
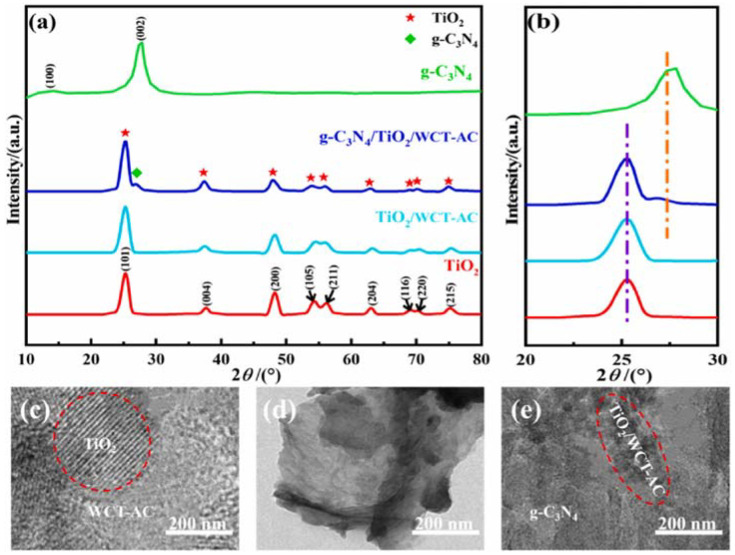
(**a**) XRD patterns of the samples. (**b**) Locally amplified XRD pattern of the sample. TEM images: (**c**) TiO_2_/WPT-AC, (**d**) g-C_3_N_4_, (**e**) g-C_3_N_4_/TiO_2_/WCT-AC. Red circles highlights the presence of TiO_2_ and TiO_2_/WCT-AC in composites. Reproduced from [90] with permission from Elsevier.

Zhang et al. prepared TiO_2_ composites by the addition of WCT-AC [90]. The authors found the peaks at 2θ of 25.3°, 37.7°, 48.0°, 53.5°, 54.7°, and 62.7° corresponding to the (101), (004), (200), (105), (211), and (204) crystal planes of rutile TiO_2_ (Figure 5a). The addition of WCT-AC did not alter the crystal structure of TiO_2_. The successful preparation of the g-C_3_N_4_/TiO_2_/WCT-AC was confirmed by the new diffraction peak observed at 2θ of 27.7°, corresponding to the (002) crystal plane of g-C_3_N_4_, compared with TiO_2_/WCT-AC and TiO_2_ alone (Figure 5b) [90].

Kaur [27] prepared graphene oxide (GO)/nitrogen (N)-doped TiO_2_ nanocomposites. XRD analysis indicated a decreased crystallite size compared with pure N-TiO_2_. This reduction is likely due to the incorporation of GO, which forms a thick coating on the surface of N-TiO_2_, hindering the growth of its crystals. A similar effect (the decreased bandgap, from 3.03 to 2.29 eV) was observed by Rojas-Guerrero, when integrating MIL-100(Fe) into TiO_2_ [92]. This reduction is related to the enhanced photocatalytic activity of the prepared materials in the visible light spectrum compared with pure TiO_2_ [27,30,92].

**Figure 6:**
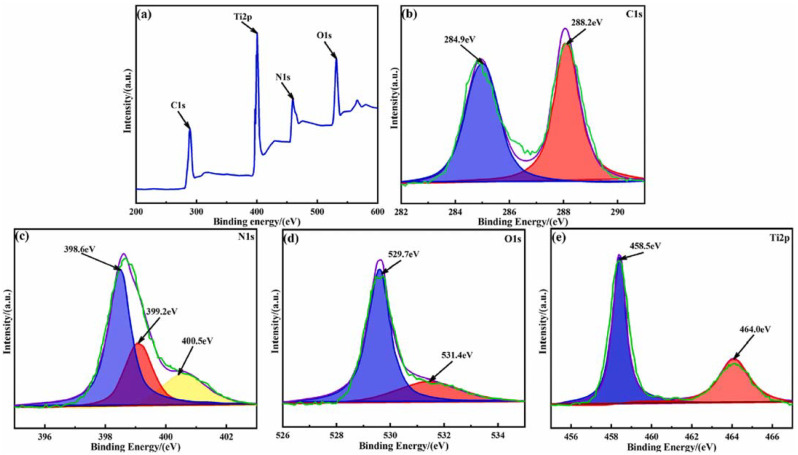
High-resolution XPS spectra of (**a**) g-C_3_N_4_/TiO_2_/WCT-AC photocatalyst material, (**b**) C 1s, (**c**) N 1s, (**d**) O 1s, and (**e**) Ti 2p. Reproduced from [90] with permission from Elsevier.

XPS analysis was conducted to evaluate the surface composition and the oxidation states of elements in the synthesized materials [30,90]. The Ti 2p spectra of the TiO_2_-based composite photocatalysts appeared in the range of 450–470 eV, showing two distinct peaks attributed to the Ti 2p_1_/_2_ and Ti 2p_3_/_2_ binding energies, which are characteristic of the Ti^4^⁺oxidation state in anatase-phase TiO_2_ [27,90,92]. Figure 6a presents the high-resolution XPS analysis results of the C 1s, N 1s, Ti 2p, and O 1s spectra for the g-C_3_N_4_/TiO_2_/WCT-AC photocatalyst [90]. In the C 1s region, peaks at 398.6 eV, 399.2 eV, and 400.5 eV correspond to sp^2^-hybridized carbon in C=C and N=C–N bonds (Figure 6b). The N 1s spectrum also shows peaks at 398.6 eV, 399.2 eV, and 400.5 eV, attributed to sp^2^-hybridized nitrogen, π–π* interactions, and the s-triazine ring structure of C=N–C bonds, respectively (Figure 6c). The O 1s spectrum displays peaks at 529.7 eV and 531.4 eV, corresponding to Ti–O bonds within the TiO_2_ lattice and surface O–H groups (Figure 6d). Lastly, the Ti 2p spectrum shows characteristic peaks at 458.5 eV and 464.0 eV, assigned to the Ti 2p_3_/_2_ and Ti 2p_1_/_2_ energy levels, respectively (Figure 6e) [90].

## 3. Performance of TiO_2_-Based Photocatalysts for MP/NP Degradation Under Visible Light Irradiation

### 3.1. Plastic Pollutant Types

**Figure 7:**
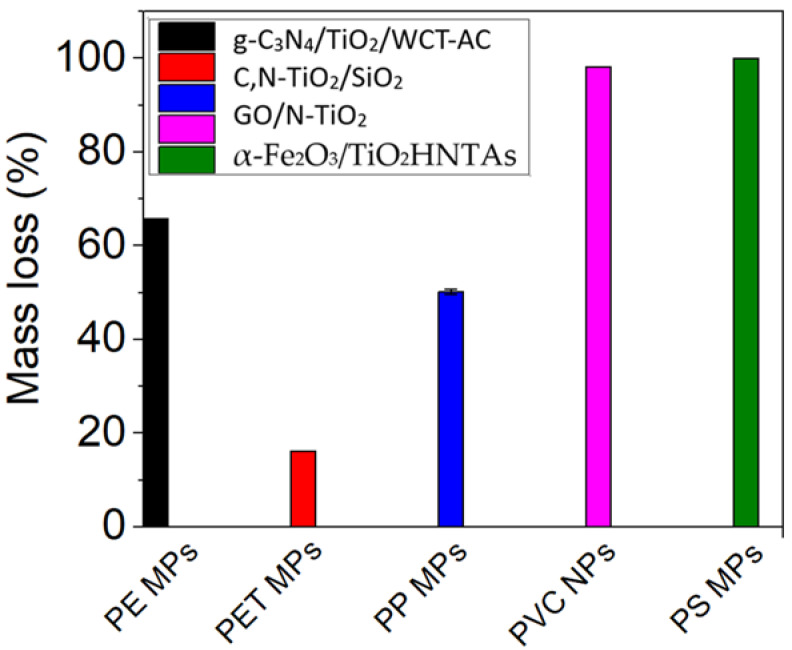
The highest mass loss % for the degradation MPs/NPs by using TiO_2_ and TiO_2_ composite photocatalysts under visible light.

Figure 7 shows the photocatalytic degradation of the MPs/NPs by estimating the highest mass loss in the presence of TiO_2_-based photocatalyst materials, according to the data from Table 1.

Various plastic pollutants have been analyzed for photocatalytic degradation under visible light using TiO_2_-based photocatalysts. These include PE MPs extracted from wastewater [90], commercially available exfoliating scrubs made of high-density polyethylene (HDPE) or mechanically fragmented black bags composed of low-density polyethylene (LDPE) [38,89,94], polyethylene terephthalate (PET) NPs derived from water bottles [92], PET MPs obtained by grinding PET food containers [93], polypropylene (PP) MPs [91], nylon 6 MPs [30], and commercially available polystyrene (PS) microspheres [95]. These are also reported as being the most common plastics found in aquatic environments.

The size dimensions of the MPs investigated in this study were ~150 µm, 814 ± 91 µm, and 382 ± 154 µm for HDPE MPs [90,94]. For LDPE, the particle sizes were (5 ± 0.01) mm × (5 ± 0.01) mm and (3 ± 0.01) mm × (3 ± 0.01) mm [94]. PET NPs were smaller than 1 μm [88], while PET MPs measured ≤500 μm [93]. PS MPs were reported to be 310 nm and within the range of 2.0–2.9 μm [95]. Polyvinyl chloride (PVC) NPs encapsulated with the fluorescent dye perylene-3,4,9,10-tetracarboxytetrabutylester (PTE) had a hydrodynamic size of 120.9–123.7 nm, a polydispersity index of 0.132, and a zeta potential ranging from −21.5 to −30.3 mV at pH 7 and were tested using a photocatalytic degradation method [27].

The investigation into the photocatalytic degradation of MPs and NPs demonstrates that these emerging plastic pollutants do not achieve complete mineralization. In contrast to conventional organic pollutants, which are soluble and more susceptible to degradation due to reactive functional groups (e.g., double bonds, aromatic rings, -OH, -NO_2_) [99,100,101,102], MPs and NPs display a significantly higher resistance to photocatalytic breakdown under visible light using modified TiO_2_-based catalysts.

This resistance is primary attributed to their morphology, chemical composition, and particle size, as well as the limited reactivity of the ROS generated by the photocatalyst materials [103]. A high molecular weight, surface hydrophobicity, the lack of reactive functional groups, and the presence of certain chemical bonds, such as esters and stable C–C bonds, in their structure reduce MP/NP susceptibility to degradation [104]. Moreover, additives like photosensitizers and antioxidants, which are designed to enhance the durability of plastics, further hinder photocatalytic degradation. At higher dimensions, the degradation efficiency is reduced. Conversely, smaller plastic particles exhibit enhanced degradation performance. This is primarily because NPs have a higher surface-area-to-volume ratio and more abundant surface functional groups, providing additional reactive sites that facilitate degradation.

### 3.2. Methods for MP/NP Degradation Evaluation

The degradation of MPs/NPs has been evaluated by measuring mass loss (according to Equation (11), turbidity, changes in the carbonyl index (CI) as an indicator of MP oxidations, and total organic carbon (TOC) [30,76,92,94]. Water-soluble degradation by-products were analyzed using gas chromatography–mass spectrometry (GC-MS) or pyrolysis (Py)-GC/MS analyses [27,29].

The carbonyl index is the ratio of absorbance at 1710 cm^−1^ (carbonyl, C=O) to 1508 cm^−1^ or 1504 cm^−1^ (aromatic, C=C) peak heights [92,93] or 1380 cm^−1^ (an internal thickness band as reference peak) [94] (FTIR analysis).

(11) $$\mathrm{Mass}\;\mathrm{loss}\;(\%)=\frac{m_{0}-m_{f}}{m_{0}}\times 100$$

where m_0_ is the initial mass of plastic pollutant (mg) and m_f_ is the final mass of plastic pollutant (mg).

The morphology and formation of cavities in the MPs/NPs structure were observed using SEM analysis [92,95].

### 3.3. Doping TiO_2_ Photocatalysts

Photodegradation performance under visible light depends on the photocatalyst material, plastic pollutant type, and experimental conditions such as pH and light intensity [104,105]. Therefore, factors such as the mass ratio of photocatalyst material components, their crystallinity, optical bandgap, and the pH of the solution significantly influence photocatalytic efficiency.

**Figure 8:**
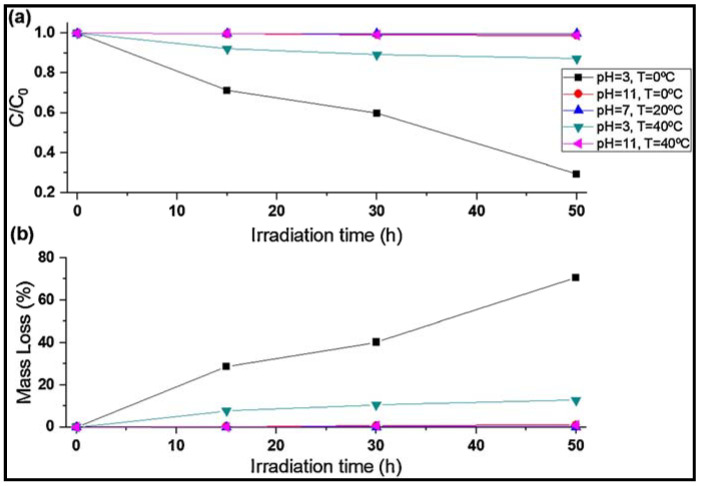
Degradation plots of MPs after photocatalytic experiments conducted at pH values of 3, 7, and 11, and temperatures of 2, 20, and 40 °C under 50 h irradiation. Degradation was expressed in terms of (**a**) initial concentration (C_0_) and final concentration (C) of MPs at specific time, and (**b**) mass loss of MPs. Reproduced from [38] with permission from Elsevier.

Ariza-Tarazona et al. [38] conducted a study for the degradation of HDPE primary MPs extracted from a commercially available facial scrub, using a C,N-TiO_2_ photocatalyst over a pH range of 3 to 11 and temperatures between 0 and 40 °C. This photocatalyst achieved the highest HDPE MP mass loss of 71.77 ± 1.88% under acidic (pH 3) and at 0 °C conditions (Figure 7). Under these experimental conditions, the HOO^•^ generation was enhanced by elevated proton concentration, while the low temperature facilitated polymer brittleness and fragmentation. In contrast, higher temperatures and alkaline pH conditions inhibited the degradation process (Figure 8). Jeyaraj et al. [91] also reported a 50% weight loss of PP MPs under solar irradiation with acidic pH conditions.

Llorente-García et al. [94] investigated the effect of MP size and shape on the photocatalytic degradation of HDPE and LDPE MPs using a mesoporous N–TiO_2_ coating. The authors reported that HDPE MPs with dimensions of 382 ± 154 µm exhibited a higher mass loss (4.65 ± 0.35%) compared with LDPE fragments with dimensions of (3 ± 0.01) mm × (3 ± 0.01) mm, which showed a mass loss of 1.38 ± 0.13%. The interaction of TiO_2_-derived ROS with the plastic surfaces revealed that HDPE’s greater surface area favored greater photocatalytic degradation than particles with larger sizes [94]. Similarly, it was reported that the reactivity of ^•^OH is higher for NPs compared with MPs, due to their smaller particle size and high surface area [92]. Also, Xue et al. [95] reported that PS MP spheres with sizes of 310 nm were completely degraded compared with those with dimensions ranging from 2.0 to 2.9 μm.

The GO/N-TiO_2_ composite with a 1:3 component ratio exhibited a high photocatalytic degradation efficiency of PVC-PTE NPs at 98.2%, compared with 59.98% achieved with pure N-TiO_2_ [27]. The enhanced photocatalytic activity was due to GO, which acted as an electron acceptor facilitating the transfer of excited electrons from the conduction band of N-TiO_2_ to GO. This transfer minimizes charge carrier recombination, thereby improving PVC-PTE NP degradation. In the absence of light, at pH 4, the removal efficiencies for PVC-PTE NPs were 51.42%, 23.17%, and 17.6%, for GO/N-TiO_2_ 1:3, GO/N-TiO_2_ 1:1, and GO/N-TiO_2_ 3:1 composites, respectively [27]. The degradation products of PVC were analyzed using GC-MS [27].

The experimental results obtained by Xue et al. [95] revealed that there is a synergistic effect between light activation and the Fenton-like (thermal catalytic) reaction under the photocatalytic degradation of PS MPs with an α-Fe_2_O_3_/TiO_2_HNTAs catalyst, which lead to an activation energy barrier.

**Figure 9:**
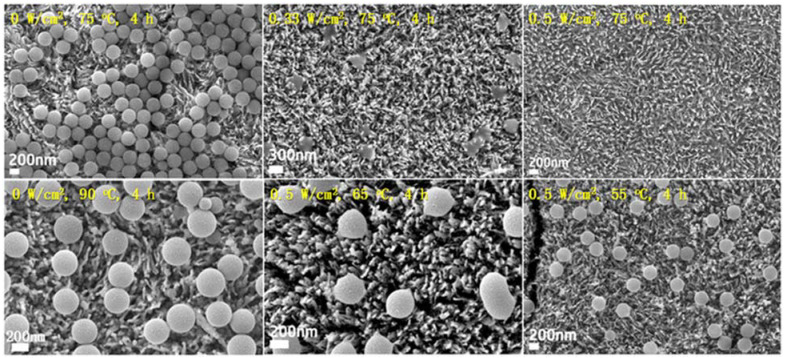
The effect of light intensity and temperature on PS degradation using α-Fe_2_O_3_/TiO_2_HNTAs catalyst. Reproduced from [95] with permission from Royal Society of Chemistry.

As shown in Figure 9, the degradation rate of PS MPs increased with higher light intensity. No degradation was observed in the absence of light (0 W cm^−^^2^). When the reaction system was heated to 90 °C in the dark, the size and shape of the PS spheres remained unchanged. However, under light irradiation at 0.33 W cm^−^^2^, noticeable melting and degradation of PS occurred. Complete mineralization (100%) was achieved when the light intensity was increased to 0.5 W cm^−^^2^ and temperature induced by light intensity was 75 °C.

#### Mechanism of MP/NP Degradation Using Doped TiO_2_

Incorporating heteroatoms into a TiO_2_ semiconductor is a commonly used strategy to introduce additional energy levels within the bandgap, thereby expanding the light absorption range.

**Figure 10:**
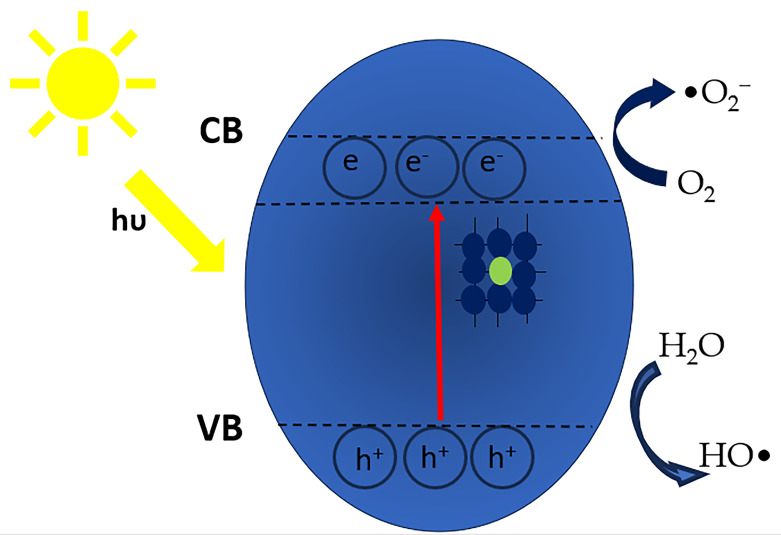
Proposed photocatalytic mechanism for doped TiO_2_ semiconductor. Adapted from [106].

When doped TiO_2_ is illuminated with visible light, its electrons become energized and transition to the conduction band (CB), leaving behind vacancies (h^+^) in the valence band (VB) of the photocatalyst material. The electrons in the CB of doped TiO_2_ can transfer to the CB of the dopant, facilitating the reduction of the O_2_ and H_2_O molecules adsorbed on its surface (Figure 10). Meanwhile, HO^−^ in the VB are oxidized by the h^+^, generating ^•^OH that break down the MPs adsorbed onto the material’s surface into smaller fragments or mineralizing them into CO_2_ and H_2_O.

A higher k value was associated with smaller particle sizes [94], as well as values of (27.4 ± 3.3) × 10^−4^ h^−1^ and an R^2^ value of 0.9315 for the N-TiO_2_ coated batch reactor [89], and (14.2 ± 3.12) × 10^−4^ h^−1^ for a mesoporous N-TiO_2_ coating [94].

### 3.4. Heterojunction with TiO_2_

**Figure 11:**
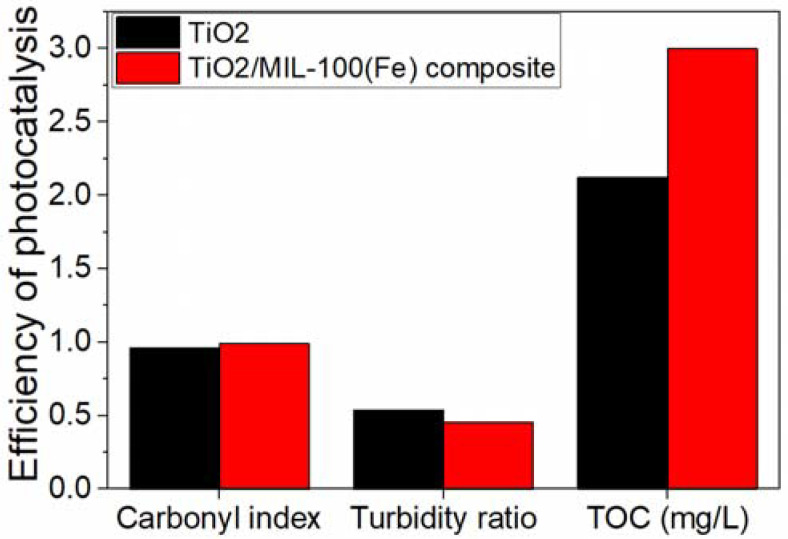
Efficiency of PET NP degradation using TiO_2_-MIL-100(Fe) composite photocatalyst compared with TiO_2_ photocatalyst under the optimum process conditions. Adapted from [92].

Photocatalytic experiments conducted in a 200 mL batch reactor demonstrated that the modified TiO_2_/MIL-100(Fe) photocatalyst outperformed TiO_2_ alone under simulated light for the degradation of PET NPs [92]. This was evidenced by an increase in the CI from 0.96 to 0.99 (FTIR analysis), a rise in TOC content from 2.12 mg/L to 3.00 mg/L, and a reduction in the turbidity ratio from 0.539 to 0.454 [92] (Figure 11).

Response surface methodology (RSM) was employed to optimize the photocatalytic degradation of PET NPs under simulated solar light by examining the effects of suspension pH and MIL-100(Fe) loading on the TiO_2_/MIL-100(Fe) catalyst [92]. The results indicated a high photocatalytic degradation efficiency of PET NPs at pH 3, attributed to the Coulombic attraction between PET NPs and the TiO_2_-MIL-100(Fe) composite surface, which is enhanced under acidic conditions.

The degradation of PET NPs was further evidenced by the formation of volatile by-products, leading to cavities in plastic pollutants, as observed using SEM analysis [92].

The photocatalytic degradation of secondary PET MPs in the presence of C,N-TiO_2_/SiO_2_ photocatalysts at pH 6 and pH 8 values ranged from 9% to 16% [93]. This enhanced photocatalytic performance of the C,N-TiO_2_/SiO_2_ photocatalyst was attributed to the presence of the Ti-O-Si bond at 956 cm^−^^1^, which lead to an increase in the surface irregularities, thereby promoting the greater trapping of photogenerated charge carriers and amplifying the activity of hydroxyl radicals.

The highest TOC response (11.012 mg/L) was achieved in the case of HKUST-1(Cu/Fe) loading into the TiO_2_ photocatalyst (at a ratio of MOF to TiO_2_ of 15:85) for the degradation of the nylon 6 MP suspension at pH 7 [30]. This enhanced performance was attributed to the formation of soluble by-products during degradation and the optimized HKUST-1(Cu/Fe) ratio using the RSM approach, which effectively suppressed e^−^/h⁺ recombination, thereby facilitating nylon 6 MP oxidation. Using GC–MS, 6-aminocaproic acid, caprolactam, butyric acid, butyramide, butyraldehyde, acetic acid, acetamide, and acetaldehyde were detected [30]. Additionally, nylon 6 MPs, which possess a positive charge during oxidation, form electrostatic interactions with the negatively charged catalyst surface at pH values above the point of zero charge (PZC), between pH 6 and 7. These interactions significantly boosted the photocatalytic degradation performance [30].

Under visible light irradiation, the g-C_3_N_4_/TiO_2_/WCT-AC photocatalytic material achieved a degradation efficiency of 67.58% for PE MPs in wastewater [90]. This photocatalyst demonstrated excellent stability over five cycles. The degradation by-products of PE MPs identified through GC-MS analysis included triacontane, ethyl octadecanoate, stearic acid, ethyl hexadecanoat, hexadecanoic acid, tetragecanoic acid, tridecanoic acid, and octadecaldehyde compounds [90].

#### Mechanism of MP/NP Degradation Using TiO_2_ Heretostructures

**Figure 12:**
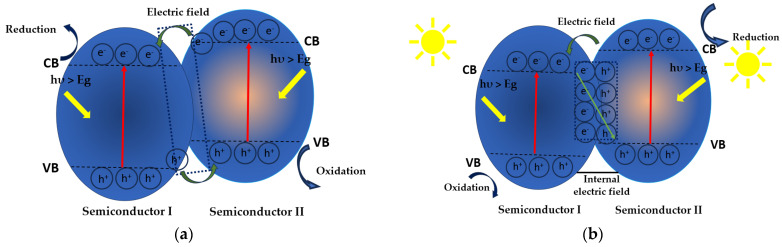
Heterojunction type II (**a**) and S-scheme (**b**). Adapted from [83].

Figure 12 displays the heterojunction type II and S-scheme for the behavior of TiO_2_-based photocatalysts containing two semiconductors during photocatalytic degradation.

The increased photocatalytic efficiency for degrading PET NPs can be attributed to a heterojunction type II mechanism, when TiO_2_ serves as semiconductor I and MIL-100(Fe) acts as semiconductor II (Figure 12a). Under simulated sunlight, e^–^ migrate from the CB of MIL-100(Fe) to the CB of TiO_2_, while photogenerated h^+^ transfer from the VB of TiO_2_ to that of MIL-100(Fe). This interaction between MIL-100(Fe) and TiO_2_ [92] facilitates charge transfer, leading to effective charge separation by reducing e^−^/h^+^ recombination. Consequently, this process significantly improves photocatalytic efficiency for degrading PET NPs.

The photocatalytic degradation of PE MPs in the presence of a g-C_3_N_4_/TiO_2_/WCT-AC composite follows an S-type charge transfer mechanism (Figure 12b). Upon visible light irradiation with energy exceeding their bandgaps, electrons in TiO_2_ and g-C_3_N_4_ are excited from stable states to higher energy levels. The photogenerated electrons in the conduction band (CB) of TiO_2_ transfer to the CB of g-C_3_N_4_, while h^+^ remain in their respective valence bands (VB), enhancing charge carrier separation and improving photocatalytic efficiency. The differences in bandgaps and Fermi levels between TiO_2_ and g-C_3_N_4_ facilitate charge transfer from g-C_3_N_4_ to TiO_2_, which lowers the Fermi level of g-C_3_N_4_ and raises that of TiO_2_. This creates a space charge region and an internal electric field (IEF) at the interface, forming an energy barrier that restricts electron migration but allows h^+^ movement. In the TiO_2_ region, band bending enables photogenerated electron release and inhibits h^+^ entry. Under visible light, these reactive species (·O_2_^−^ and ·OH) attack PE MPs, breaking them down into small inorganic compounds such as H_2_O and CO_2_. Additionally, photogenerated holes (h^+^) can directly oxidize PE MPs, further facilitating their degradation and removal.

### 3.5. Inhibition of Reactive Species

The quenching of reactive species involved in the photocatalytic degradation of plastic pollutants was investigated using different reagents: *t*-butanol (*t*-BuOH), sodium oxalate, and triethanolamine (TEA) to inhibit HO^•^ [27,90,92]; 2-propanol (2-PrOH) to inhibit both HO^•^ and h^+^ [92]; isopropanol (IPA) to inhibit h^+^ [27,90]; and *p*-benzoquinone (*p*-BQ) to inhibit ^•^O_2_^−^ [27,90]. *t*-BuOH was also used to evaluate the reactive oxygen species ^•^OH and ^•^O_2_^−^ while formic acid was used to inhibit h^+^ [30]. The results showed that ^•^OH and h^+^ are the primary reactive species responsible for the photocatalytic degradation of PET NPs and PVC-NPs by the TiO_2_/MIL-100(Fe) and GO/N-TiO_2_ composites, respectively, under simulated sunlight. In contrast, *p*-BQ and TEA were more effective in inhibiting ^•^O_2_^−^ and HO^•^ during the photocatalytic degradation of PE MPs in the presence of g-C_3_N_4_/TiO_2_/WCT-AC material.

## 4. Challenges

This review highlights that, in most cases, the current photocatalytic method conducted with TiO_2_-based composites achieves only the partial degradation of MPs and NPs at the laboratory scale, and their practical effectiveness remains limited.

Several challenges associated with photocatalytic degradation under visible light have been identified.

Firstly, the formation of minuscule and potentially harmful metabolites introduces new pollution concerns. The long-term effect of degradation products, such as monomers and oligomers, must be carefully evaluated to ensure that the by-products generated during this process do not pose ecological or health risks. The identification and quantification of these by-products are necessary for assessing their potential toxicity [29]. Comprehensive environmental impact assessments, including life cycle assessments (LCAs) and detailed studies of intermediate degradation products and nanoparticle release, are vital for ensuring the environmental sustainability of this approach [29]. Integrating membrane separation with photocatalytic processes could offer an effective approach for the complete removal of MPs [29].

Secondly, TiO_2_-based photocatalyst materials, when used as nanopowders in water systems, can often lead to unintended secondary pollution. This includes residual nano-photocatalyst particles, ion leaching, free radicals, and toxic by-products, which pose threats to aquatic organisms and may endanger human health [106]. When used in slurry form, TiO_2_ particles must be separated after treatment, necessitating additional filtration or sedimentation steps. It is essential to evaluate the potential ecological and health consequences of TiO_2_ composites after their use, as their release into ecosystems may present issues that were not fully explored in this study. Although TiO_2_ is widely regarded as a material with low toxicity, concerns have arisen due to its designation by the International Agency for Research on Cancer (IARC) as a possible human carcinogen [107]. Prolonged exposure to TiO_2_ NPs may lead to their accumulation in tissues, possibly causing chronic illnesses and raising significant concerns about their detrimental health impacts. Once thought to be biologically inert, TiO_2_ NPs have been shown in numerous in vitro studies to possess cytotoxic and genotoxic characteristics. These effects are associated with the generation of OH^●^, HO_2_^●^, and O_2_^●−^ active species (ROS) and the activation of molecular pathways linked to inflammation and cellular damage. Furthermore, in vivo research has demonstrated that TiO_2_ NPs, once introduced into the circulatory system, can accumulate in vital organs, resulting in harmful effects.

Thirdly, according to the limited performance of TiO_2-_based photocatalysts to degrade MPs and NPs, future studies are necessary [108]. Various strategies have been employed to increase the performance and recyclability of TiO_2-_based photocatalysts [76]. For instance, bimetallic HKUST-1 (Cu/Fe)-derived CuO/TiO_2_ composites were supported on glass substrates [30], while TiO_2_/MIL-100(Fe) composites were immobilized on mineral perlite and placed within fine mesh stainless steel baskets [92] prior to the photocatalytic process. Zhang et al. [90] investigated the recycling stability of a TiO_2_-based photocatalyst.

Fourthly, detecting NPs using conventional spectrophotometric techniques is challenging. To address this issue, encapsulating NPs may serve as an effective strategy. For instance, PVC NPs have been encapsulated in perylene tetrabutylester (PTE) [27]. Given that the concentrations of MPs in water typically ranged from 0.02 to 0.03 mg/L, it is essential to employ methods that concentrate these particles for more accurate analysis.

Fifthly, the photocatalytic degradation of MPs and NPs using TiO_2_ composites is typically conducted under controlled laboratory conditions, which may not accurately reflect the dynamics of natural ecosystems or large-scale wastewater treatment processes (WWTPs). In practical applications, plastics are often combined with other substances, complicating the ability of photocatalysts tailored to a specific polymer to function efficiently.

In future studies, the design of multi-functional photocatalysts capable of operating under sunlight should focus on shifting from degrading a single type of polymer to effectively breaking down a wide variety of polymeric materials.

A novel approach for removing MPs, at the same time as their collection from marine environments, involves a floating photoreactor equipped with photocatalysts. This device, which can be towed by a boat navigating the ocean, effectively removes MPs from the water’s surface by leveraging abundant solar energy [64].

Photocatalysis, as an advanced water treatment method, can be integrated into the tertiary stage of WWTPs and MPs can be decomposed into CO_2_ and H_2_O or converted into molecular by-products that are potentially less harmful or degrade more rapidly [93]. Nevertheless, to address its drawbacks, such as slow reaction rates and reliance on light, photocatalysis is most effective when combined with complementary methods, such as filtration for preliminary or final treatment.

## 5. Conclusions

The degradation of microplastics (MPs) and nanoplastics (NPs) has become a critical focus in environmental remediation due to the widespread accumulation of plastic pollutants in key ecosystems such as aquatic systems, soil, and the atmosphere.

Conventional treatment methods including filtration, coagulation–flocculation, and sedimentation remain the primary approaches for removing these plastic contaminants. However, these techniques often fail to fully eliminate smaller plastic particles, highlighting the need for more advanced alternatives.

The literature provides limited data on the degradation of MPs and NPs using TiO_2_ composites-based photocatalysis under light irradiation. To enhance photocatalytic efficiency within the visible light spectrum, researchers have employed strategies such as bandgap engineering through doping, heterojunctions formation, and surface modification.

The key factors influencing photocatalytic performance include the catalyst’s structural properties, the physicochemical characteristics of the target plastic pollutants, and optimal operational parameters such as light intensity and the pH of the solution.

The findings highlight the superior performance of certain photocatalysts, such as α-Fe_2_O_3_/TiO_2_HNTAs, HKUST-1(Cu/Fe)-derived CuO/TiO_2_ (TCFH), HKUST-1(Cu/Fe)-derived CuO/TiO_2_ (TCFH), and C,N-TiO_2_ composites. These composites offer a high porosity and stability, which contribute to more efficient degradation of MPs and NPs.

The degradation behavior varies significantly among different MPs and NPs. In this review, the degradation efficiency measured by the mass loss or total organic carbon (TOC) follows the order: PS > nylon 6 > PVC > PE > PP > PET. Smaller plastic particles tend to degrade more readily under photocatalytic conditions. To improve the degradation outcomes, heterojunction and S-scheme systems that combine the advantages of multiple photocatalytic materials have shown promise.

Despite recent advances, further research is needed to develop next-generation TiO_2_-based photocatalyst composites, optimize photocatalytic conditions, and address scalability challenges. These efforts are essential for achieving the efficient and complete degradation of small plastic pollutants in real-world environmental settings, given the vast quantities of plastic products polluting our vital ecosystems.
